# Pediatric Hemispheric High-Grade Gliomas and H3.3-G34 Mutation: A Review of the Literature on Biological Features and New Therapeutic Strategies

**DOI:** 10.3390/genes15081038

**Published:** 2024-08-06

**Authors:** Marta Bonada, Matilde Pittarello, Emerson De Fazio, Alessandro Gans, Paolo Alimonti, Hasan Slika, Federico Legnani, Francesco Di Meco, Betty Tyler

**Affiliations:** 1 Department of Neurosurgery, Fondazione IRCCS Istituto Neurologico Carlo Besta, Via Celoria 11, 20133 Milan, Italy; marta.bonada@edu.unito.it (M.B.); federico.legnani@gmail.com (F.L.); francesco.dimeco@istituto-besta.it (F.D.M.); 2 Department of Oncology and Hemato-Oncology, University of Milan School of Medicine, Via Rudini 8, 20122 Milan, Italy; alessandro.gans@unimi.it; 3 Department of Biomedical Sciences, Humanitas University, 20072 Milan, Italy; matilde.pittarello@gmail.com; 4 Department of Medicine, Vita-Salute San Raffaele University School of Medicine, 20132 Milan, Italy; e.defazio@studenti.unisr.it; 5 ASST Ovest Milanese, Neurology and Stroke Unit, Neuroscience Department, 20025 Legnano, Italy; 6 Department of Neurosurgery, Brigham and Women’s Hospital, Harvard Medical School, Boston, MA 02120, USA; p.alimonti@studenti.unisr.it; 7 Department of Neurosurgery, Johns Hopkins University School of Medicine, Baltimore, MD 21231, USA; hslika1@jhmi.edu

**Keywords:** glioma, high-grade, histone mutation, immunotherapy, pediatric, targeted therapy

## Abstract

Pediatric high-grade glioma (pHGG) encompasses a wide range of gliomas with different genomic, epigenomic, and transcriptomic features. Almost 50% of pHGGs present a mutation in genes coding for histone 3, including the subtype harboring the H3.3-G34 mutation. In this context, histone mutations are frequently associated with mutations in *TP53* and *ATRX*, along with *PDGFRA* and *NOTCH2NL* amplifications. Moreover, the H3.3-G34 histone mutation induces epigenetic changes in immune-related genes and exerts modulatory functions on the microenvironment. Also, the functionality of the blood–brain barrier (BBB) has an impact on treatment response. The prognosis remains poor with conventional treatments, thus eliciting the investigation of additional and alternative therapies. Promising molecular targets include *PDGFRA* amplification, *BRAF* mutation, *EGFR* amplification, *NF1* loss, and *IDH* mutation. Considering that pHGGs harboring the H3.3-G34R mutation appear to be more susceptible to immunotherapies (ITs), different options have been recently explored, including immune checkpoint inhibitors, antibody mediated IT, and Car-T cells. This review aims to summarize the knowledge concerning cancer biology and cancer-immune cell interaction in this set of pediatric gliomas, with a focus on possible therapeutic options.

## 1. Introduction

The most common solid neoplasms in childhood are represented by tumors of the central nervous system (CNS), with histological diagnosis of glioma in approximately 50% of cases [1]. Differently from adults, high-grade gliomas (HGGs) are less frequent in children, as they more often present with low-grade gliomas (LGGs) [2,3]. Molecular features of pHGG significantly differ from the adult form, even if they are morphologically comparable. For this reason, innovative therapeutic strategies discovered and assessed for adult gliomas did not provide impactful results in the pediatric population [4]. Notable molecular differences also exist within pHGGs, allowing for different subtypes to be defined according to genomic, epigenomic, and transcriptomic profiles. Almost 50% of pHGGs fall into the subgroup characterized by the mutation of genes encoding for histone H3 [4]. These mutations affect gene expression, DNA repair, and cell replication since histones play an essential role in the condensation status of DNA [5]. These alterations are especially represented by the K27M and G34V mutations that lead to tumorigenesis [6].

Diffuse hemispheric gliomas (DHG) with “glycine 34-to-arginine/valine substitutions in histone gene 3.3” (H3.3-G34-mutant) are aggressive primary brain tumors originating in the cerebral hemispheres, prevalently in the temporo-parietal lobes [7,8,9,10]. Occurring almost exclusively in the adolescent and young adult population, with a peak incidence at 15 years of age, DHGs bear a dismal prognosis with a median overall survival (mOS) of around 18 months [6,11]. DHGs were first included in the World Health Organization classification of Central Nervous System Neoplasms in 2021 (WHO 2021) [12], and their current understanding is based on a circumscript body of scientific literature. While these studies have defined a particular mutation pattern, phylogeny, and cell biology for these tumors, there is still a lack of understanding about the immune tumor microenvironment (TME) and its implications for developing immunotherapy options for H3.3-G34-mutant glioma patients. This review aims to summarize the knowledge concerning cancer biology and cancer-immune cell interaction in this set of pediatric gliomas, highlighting the reported and ongoing therapeutic efforts to harness immunotherapy options for these patients.

## 2. Brain Cancer Biology: Genetic and Epigenetic Features

**Figure 1:**
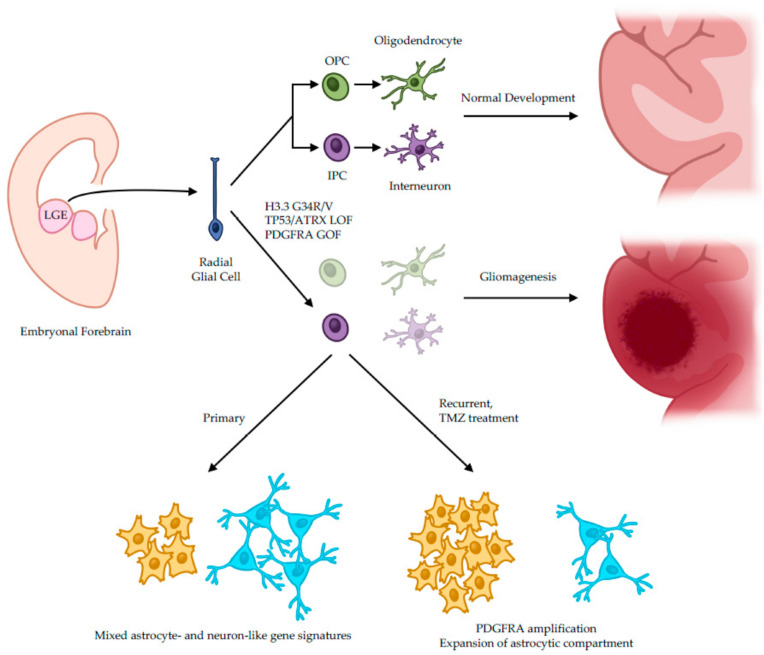
Developmental chain of normal brain vs. H3.3-G34-mutant glioma. OPC: oligodendrocyte progenitor cell; IPC: interneuron progenitor cell; LOF: loss of function; GOF: gain of function; TMZ: temozolomide.

In 2020, Chen et al. [9] performed a thorough bulk and single-cell genomic, epigenomic, and transcriptomic profiling of H3.3-G34-mutant glioma samples from 95 patients. Their results are summarized in Figure 1. Mutation analysis revealed *TP53* and *ATRX* mutations in 95% and 84% of cases, respectively, along with *PDGFRA* amplifications in 44% of cases at diagnosis and 81% of recurrent tumors. Among the recurrent cases, 85% of *PDGFRA* amplifications occurred in the second-to-fourth section of the extracellular (EC) Ig-like domain, leading to constitutive activation of the downstream MAPK-ERK signaling pathway. Furthermore, single-cell RNA sequencing (scRNAseq) on tumor samples traced the cellular origin of H3.3-G34-mutant gliomas to early cortical interneuron progenitors (CIPs) present during fetal neurodevelopment. This peculiar cell population arises from ventral radial glial cells (vRGCs), an early neural stem cell compartment forming structures of the ventral forebrain known as ganglionic eminences, as demonstrated by the retention of the marker genes *GSX2* and *DLX1/2* in patient samples [10]. Furthermore, as CIPs are responsible for generations of future cortical interneurons and oligodendrocytes, tumor cells from H3.3-G34-mutant gliomas display dual astrocytic and neuronal cell states and gene signatures, along with expanded neuron-glial compartments in histological sections, in the absence of oligodendrocyte lineage representation.

Downstream analysis revealed the specific mutation mechanisms responsible for gliomagenesis in these tumors. Specifically, oncohistone H3.3-G34R/V mutations stall CIP differentiation in its early phases, keeping an open chromatin state at the *GSX2* enhancer site. This event triggers an opportunity for co-option by the nearby *PDGFRA* promoter through a chromatin looping mechanism and subsequent enhancer hijacking.

Interestingly, in models of tumor recurrence post-temozolomide treatment, there was a strong selective pressure towards *PDGFRA* amplification. This phenomenon caused downstream MAP-ERK pathway overactivation [13] and consequent expansion of the astrocytic compartment [9]. Together, these findings highlight crucial pathogenetic mechanisms at the base of H3.3-G34-mutant glioma onset and bear important prognostic and therapeutic implications for small molecule inhibitor testing in H3.3-G34-mutant gliomas.

Subsequent work relied on preclinical models of H3.3-G34-mutant gliomas to demonstrate their peculiar developmental origin and regional specificity. Bressan and colleagues [14] engineered neural stem cells (NSCs) from the fetal forebrain and hindbrain. They selected ventral forebrain NSCs using a combination of marker genes including *FOXG1*, *DLX2/6*, and *EMX2*, whose expression was also enriched in forebrain derivate structures like the neocortex and striatum and their related NSCs.

Chromatin immunoprecipitation and sequencing (CHIP-seq) analysis showed that H3.3-G34 mutations in forebrain NSCs do not induce widespread epigenetic alterations by changing histone mark deposition or H3.3 distribution patterns, such as those determined by H3.3-K27M oncohistone mutations in hindbrain NSCs. Instead, they seem to act on genes already expressed in forebrain progenitors. At the molecular level, H3.3-G34R mutation prevents binding of ZMYND11, a H3.3 K36me3 transcriptional co-repressor, to key forebrain regulator genes *DMRTA2* and *ARX*. This lost downregulation boosts forebrain gene expression in H3.3-G34-mutant glioma cells. Other studies have linked the epigenetic H3.3 K36me3 alteration pattern to increased *MYC* expression in H3.3-G34R/V gliomas [15,16,17]. Another forebrain identity master regulator, *FOXG1*, is expressed in pHGG cells independently of the G34R mutation. Crispr-Cas9 downregulation ablated tumor formation in H3.3-G34R-mutant gliomas but not in H3.3-wildtype adult hemispheric HGGs [14]. Finally, the authors showed that concomitant *TP53* deletion and PDGFRA overexpression blocked hindbrain NSCs while inducing neocortical NSC proliferation [14]. Together, these findings show that H3.3-G34-mutations may induce gliomagenesis exclusively in forebrain NSCs, thereby hinting toward strong specificity for this cellular niche.

In another study, Funato et al. [18] developed a protocol to model H3.3-G34-mutant pHGGs from human embryonic stem cells (hESCs). First, combined H3.3-G34R constitutive expression and double *TP53/ATRX* Crispr-Cas9-based knockout (KO) were induced in hESCs, followed by separate in vitro differentiation into neural progenitor cells of the ventral forebrain (vFNPCs) or ventral hindbrain (VHNPCs). Of the two resulting cell lines, only vFNPCs developed a tumorigenic phenotype, thereby proving the unique susceptibility of the ventral forebrain to the mutations that offset pHGGs. Further analysis showed aberrant splicing events, such as ZMYND11-mediated intron retention at the *NOTCH2NL* gene locus resulting in *NOTCH2NL* amplification. This finding was especially relevant since *NOTCH2NL* favors cortical progenitor cell proliferation during neurodevelopment. With the H3.3-G34R, *TP53*, and *ATRX* triplet of mutations, *NOTCH2NL* amplification in tumor cells boosts glioma cell proliferation. *NOTCH2NL* overexpression resulted in a two-fold increase in forebrain cell growth specifically, while shRNA *NOTCH2NL* knockdown in H3.3-G34R tumor cell lines reduced their growth, capacity for spheroid formation in vitro, and tumor formation upon in vivo implantation. These findings suggest that *NOTCH2NL* is a critical marker of forebrain cell identity and a pHGG proliferation driver.

Overall, these studies demonstrate that a distinct set of mutations, including the H3.3-G34R/V oncohistone mutations, *TP53* and *ATRX* loss of function, and PDGFRA amplification, underlie H3.3-G34-mutant gliomagenesis. Evidence suggests that oncohistone mutations have reduced tumorigenicity and are not essential for tumor maintenance [10,14,15,19,20] Thus, gliomagenesis only ensues when such mutations concur in a restricted cellular compartment of the developing brain, namely the forebrain CIPs. One peculiar epigenetic aberration present in these tumors is the loss of the H3.3-K36 trimethylation (H3.3-K36me3) pattern [14,15,16,17]. Lysine methyltransferases performing H3.3-K36 deposition share the SET domain, which recognizes H3.3-K36 residues through a narrow structural channel. The substitution of glycine into bulkier amino acids such as arginine or valine at H3.3, the typical mutation of H3.3-G34R/V-mutant gliomas, impairs the entry of the H3.3 histone tail into the SET catalytic domain, preventing trimethylation [10,15]. H3.3K36me3 loss impairs neuronal differentiation and stalls tumor cells in a dual astrocytic-neuron state resembling forebrain CIPs. This block in differentiation allows tumor cells to maintain an open chromatin landscape at enhancers such as *GSX2*, which are then hijacked by the overexpressed *PDGFRA* gene [9].

Other downstream effects of H3.3-G34R/V mutations include overexpression of marker genes for forebrain identity and aberrant splicing of genes crucial for neurodevelopment, which act as additional drivers of tumor cell growth [14,18]. These findings highlight a peculiar molecular interplay between tumor-initiating mutations and their associated epigenetic aberrations in H3.3-G34R/V gliomas.

### The Tumor Microenvironment in pHGG

Besides the effects on tumor cells, H3.3-G34 oncohistone mutations exert modulatory functions on the immune microenvironment of pHGG. By leveraging a combination of RNA-seq and CHIP-seq in an engineered immunocompetent mouse model, Garcia-Fabiani et al. [21] demonstrated that the H3.3-G34R has an impact on the expression of genes from the JAK-STAT and “type I interferon” (IFN) pathways, finally causing their overexpression. Moreover, H3.3-G34-mutant tumor cells exhibit aberrant chromatin regulation at the gene loci for several pro-inflammatory cytokines, leading to a more permissive TME. In this context, immune cell infiltration analysis revealed increased proportions of activated CD4+ and CD8+ T cells and higher Treg/CD8+ T cell ratios in the TME of H3.3-G34R/V tumors compared to H3.3 wild-type pHGGs. Furthermore, compared to H3.3-wild-type glioma models, H3.3-G34-mutant glioma models showed depletion of monocytes and polymorphonuclear (PMN) cell populations, as well as an increase in dendritic cells (DC) and macrophages. Critically, while anti-inflammatory (M2) macrophage proportions were similar in the two models, H3.3-G34-mutant tumors exhibited an increased proportion of pro-inflammatory macrophages (M1). Finally, the authors demonstrate a boosted anti-tumor immune response following adenovirus-based TK/Fit3L gene therapy in H3.3-G34-glioma models [21]. These findings indicate that oncohistone mutations in H3.3-G34-mutant glioma cells induce epigenetic changes in immune-related genes, leading to a more immune-stimulatory TME that could be exploited for immunotherapy. 

Most studies have focused primarily on the mutation patterns and epigenetic dysregulations inducing H3.3-G34-mutant gliomagenesis. Although limited, such findings have contributed to our current knowledge of the molecular mechanisms of tumor development. Moreover, epigenetic alterations might influence the composition of immune TME, deserving further characterization and possibly representing a promising therapeutic avenue for these tumors.

Pediatric tumors driven by oncohistone dysregulations, such as undifferentiated sarcomas and diffuse midline glioma (DMG), H3.3-K27M-altered, still represent a therapeutic challenge given the poor outcomes of their current management [22]. Oncohistone-targeting agents are urgently needed to improve the prognostic outcomes for patients with these tumors, and studies are ongoing. One example is provided by Grassl and colleagues [23], reporting on H3-K27M-targeting vaccine use in eight adult patients diagnosed with DMG, an invasive and lethal form of brainstem oncohistone-driven glioma. Among these patients, five received combined treatment with PD-1 inhibitor immunotherapy. This treatment was safe, inducing a strong CD4+ T cell-mediated immune response, independent of anti-PD-1 immunotherapy, both in peripheral blood and at the tumor site. Particularly, CD4+ tumor-infiltrating cells displayed an HLA-DR MHC class II phenotype and interacted with “ionized calcium-binding adaptor molecule 1” (IBA1)-positive antigen-presenting cells (APCs). The median progression-free survival and overall survival (mPFS and mOS), respectively, were 6.2 months and 12.8 months. One patient with a strong T-cell-mediated response to the oncohistone epitope displayed complete tumor remission for more than 31 months. These findings are extremely encouraging in the treatment of DMG and pave the way for similar therapeutic efforts against other oncohistone-driven tumors, such as H3.3-G34-mutant glioma. Future studies should prioritize developing effective strategies targeting oncohistone-driven gliomas, including overcoming the blood–brain barrier and determining routes of administration. Combining new strategies with standard treatment options such as surgery and chemoradiotherapy may also be beneficial.

Radial glial cells compose the Lateral Ganglionic Eminences (LGE) of the forebrain, which arise during embryonic development. They differentiate into cortical neural progenitor cells, including OPCs (oligodendrocyte progenitor cells) and IPCs (interneuron progenitor cells), which in turn generate oligodendrocytes and interneurons, forming the postnatal telencephalon.

Alternatively, in the presence of H3.3-G34 oncohistone mutations, along with *TP53* and *ATRX* loss of function (LOF) and *PDGFRA* gain of function (GOF), H3.3-G34-mutant gliomas develop. At the transcriptional level, these tumors are characterized by astrocytic and neuronal gene signatures, which shift in favor of the astrocyte compartment at recurrence, thanks to temozolomide (TMZ)-induced *PDGFRA* amplification events.

## 3. Blood–Brain Barrier: Its Function and Role in the Setting of Pediatric Glioma

### 3.1. Function and Anatomy of the Blood–Brain Barrier

The Blood–Brain Barrier (BBB) is a dynamic, semi-permeable, tight endothelial membrane encompassing CNS microvasculature, forming the functional unit called the neurovascular unit (NVU) [24,25,26,27]. The various roles of the BBB include maintaining an adequate ionic balance for proper synaptic function, keeping the central and peripheral transmitter pools separate to avoid crosstalk, preventing macromolecule entrance in the brain compartment, protecting the brain from circulating toxins and drugs, and distributing water-soluble nutrients [27].

Endothelial cells, astrocytes, pericytes, and junctional complexes form the BBB’s foundation [28,29]. The BBB endothelium strictly regulates molecule flow between the blood and brain parenchyma. First, cells are fastened by tight junctions (TJs) and adherent junctions (AJs). Secondly, cell membranes lack fenestration and exhibit distinctive electrical properties, limiting polar molecules and cell influx. TJs, also known as zonulae occludentes, seal interendothelial clefts, preventing lateral diffusion and maintaining endothelial cell polarization. AJs are closely interconnected with the cellular cytoskeleton, similarly to TJs, and through the relationship between specific proteins such as catenins, scaffold proteins, and cadherins, they form membrane microdomains necessary for both structural purposes and inter-endothelial connections. Astrocytes, or astroglia, are morphologically complex and polarized cells distinct in the protoplasmic and fibrous subtypes. Astrocyte endfeet reach the basement membrane and form the glia limitans externa through specific proteins such as aquaporin-IV (AQP4) and the dystroglycan-dystrophin complex. Astrocytes are crucial for the immune response and maintaining local homeostasis. Pericytes are mural cells arranged along the microvasculature walls in close communication with endothelial cells via *PDFG-B* signaling. Such cells are involved in adjusting cerebral blood flow and modulating angiogenesis and neuroinflammation [26,27,30].

### 3.2. Transport across the Blood–Brain Barrier

Transport across the BBB can occur through passive diffusion, active efflux, carrier-mediated transport (CMT), and receptor-mediated transcytosis (RMT) [31].

#### 3.2.1. Passive Diffusion

Passive diffusion mainly involves small liposoluble molecules [32] that cross the BBB through pores that form transitorily in the phospholipid bilayer [33,34]. The size of these pores restricts the passage of molecules larger than the pore. Indeed, increasing the surface area of a drug, for instance, from 52 Å^2^ (as seen in a drug with a molecular weight of 200 Da) to 105 Å^2^ (as observed in a drug with a molecular weight of 450 Da), leads to a significant reduction in its permeability across the BBB [35]. Furthermore, molecular weight determines whether a molecule can undergo passive diffusion through the BBB. Indeed, when the molecular weight exceeds 400 Da, the permeability of the drug does not increase proportionally to its lipid solubility [35]. Finally, a polar surface area over 80 Å and the presence of more than six hydrogen bonds significantly limit the passive diffusion of molecules through the BBB [36].

#### 3.2.2. Active Efflux

Active efflux of molecules is mediated by ATP-binding cassette (ABC) proteins, which are highly expressed within the CNS and limit the permeability of the BBB to toxins and therapeutic molecules [37]. The main ABC proteins include Breast Cancer Resistance Protein (BRCP, ABCG2) and P-glycoprotein (Pgp—Multidrug Resistance Protein ABCB1), both expressed on the luminal side of the BBB, and the Multidrug Resistance-associated Proteins (MRPs, ABCC1, 2, 4, 5, and possibly 3 and 6), which are located in the luminal or abluminal membrane [38,39,40]. The ABC transporter superfamily prevents several compounds from entering the brain, including drugs that target aberrant signaling pathways in glioma [41].

#### 3.2.3. Carrier-Mediated Transport (CMT)

The gene family of the Solute Carrier (SLC) Transporter includes up to 300 genes encoding for membrane-bound proteins that regulate CMT conveying different substances [42], including amino acids, carbohydrates, fatty acids, monocarboxylic acids, hormones, choline, vitamins, organic amines, nucleotides, and organic anions [31].

#### 3.2.4. Receptor-Mediated Transport (RMT)

Larger peptides cannot cross the BBB employing CMT [43]; instead, they cross the BBB by RMT [44]. These larger molecules exploit transcytosis, an endocytotic mechanism, to cross the BBB [31]. Transcytosis can occur through two types of vesicular transport: “receptor-mediated transcytosis” (RMT) and “absorptive-mediated transcytosis” (AMT) [27,31]. By RMT, large molecules can be transported across the BBB. The transport of molecules through receptor-mediated mechanisms can be exploited by developing so-called Molecular Trojan Horses. These genetically engineered proteins can exploit RMT to cross the BBB and thus deliver therapeutic molecules directly into the brain [31,45].

### 3.3. Neurofluids and Brain Tumor

The lack of clear compartmentalization within the CNS implies a dynamic relationship between the TME, brain parenchyma, and neurofluids. Neurofluid dynamics, primarily studied in neurodegeneration and neuroinflammation, have seen limited focus on primary brain tumors [46].

Space-occupying lesions (SOLs) like gliomas cause an allosteric shift in flow dynamics to maintain intracranial pressure, as per the Monro-Kellie hypothesis [47]. Gliomas invade and compress surrounding structures, altering fluid dynamics and secreting molecules that promote tumor invasion via a process called “autologous chemotaxis”. Increased interstitial fluid (ISF) flow and shear stress promote extracellular matrix degradation by MMP-2 and MMP-9, facilitating tumor invasion [48]. The intimate link between ISF and cerebrospinal fluid (CSF), illustrated by the glymphatic system and “intramural periarterial drainage” (IPAD) hypotheses, highlights the role of cerebrospinal fluid (CSF) dynamics in tumor progression [49]. Neuroimaging studies of gliomas show reduced peritumoral perivascular diffusivity, indicating glymphatic dysfunction and neuroinflammation [50,51]. This dysfunction leads to protein and toxic molecule accumulation, promoting tumor growth, neuroinflammation, and neurodegeneration [52,53]. SOLs also disrupt CSF-lymphatic flow, affecting brain immune surveillance [54]. In 2020, Song and colleagues published a glioblastoma mouse model receiving “vascular endothelial growth factor C” (VEGF-C) treatment. VEGF-C treatment in a glioblastoma mouse model improved CSF-lymphatic flow and enhanced CD8+ T-cell activity against tumors [55]. The “subarachnoid lymphatic-like membrane” (SLYM), or fourth meninges, splits the subarachnoid space into two functional compartments by guiding CSF flow dynamics and participates in bridging the cerebral structures to the dural meningeal immune system and the cranial theca.

It is worth considering whether SLYM may also play a role in the progression of brain tumors, such as gliomas and pHGGs. Thus, considering the intimate connection between the immune system and brain fluid dynamics, both disrupted within gliomas and brain tumors, it becomes necessary to develop therapeutic approaches and studies addressing these two fields synergistically.

### 3.4. The Blood–Brain Tumor Barrier

When discussing brain tumors, the BBB is often called the “blood-brain tumor barrier” (BBTB). The BBTB appears to be permeable and “leaky” [56,57], likely due to NVU dysfunction [58,59]. This leakiness is also associated with the downregulation of interendothelial TJs [60], induced by VEGF and other cytokines secreted by astrocytomas and other brain tumors [31,61]. Other hypotheses include loss of claudin1 and claudin-5 downregulation, as seen in glioblastoma [62], and loss of 55 kDa occludin, as seen in metastatic adenocarcinoma and astrocytoma [63]. Another key molecule involved in BBTB disruption is AQP4, a water channel that is upregulated both in astrocytoma and brain metastases. Its overexpression and depolarization correlate with radiological findings associated with BBB disruption [64]. In clinical practice, gadolinium enhancement on “contrast-enhanced magnetic resonance imaging” (CE-MRI) indicates BBB dysfunction [65,66].

### 3.5. Blood–Brain Barrier in the Pediatric Age

At birth, the BBB’s functionality is already established [67,68,69]. Throughout the phases of prenatal and postnatal development, the barrier mechanisms undergo dynamic modulation to create the optimal microenvironment required for the maturing brain [70]. For example, the BBB of neonates is more permissive for the entrance of amino acids in order to nourish the developing brain. This increased permeability to amino acids declines during adulthood [71].

### 3.6. Blood–Brain Barrier Heterogeneity in Pediatric Brain Tumors

In the context of pediatric brain tumors, there is a wide heterogeneity when it comes to BBTB function, as not all tumors impact the functionality of the BBB in the same way [72,73,74,75]. A study by Hong et al. [76] compared three types of pediatric brain tumors and how they affect the functionality of the BBB. The three tumor types examined were pilocytic astrocytoma, low-grade diffuse astrocytoma, and medulloblastoma (MB). The results highlighted a disruption of the BBB in medulloblastoma and pilocytic astrocytoma, as evidenced by the classical gadolinium enhancement at CE-MRI but not in the low-grade diffuse astrocytoma. In pilocytic astrocytoma, BBB alteration appeared to originate from the dysfunctional tumor cells, which could not support the BBB. Meanwhile, in medulloblastoma, the BBB alteration occurred because astrocytes could not occupy the densely cellular parenchyma of the tumor. In both cases, there was a disruption of the crucial relationship between the astrocytic foot processes and the endothelial cells, which are both critical components of the BBB [56,76].

Medulloblastoma is the most common malignant pediatric embryonic brain tumor that develops within the cerebellum. These tumors are subdivided into four molecular subtypes: Wingless (WNT), Sonic Hedgehog (SHH), Group 3 (Gp3), and Group 4 (Gp4) [77]. Regarding BBB breakdown, there appears to be heterogeneity within the different medulloblastoma subtypes as well [65]. The presence of enhancement in WNT MB indicates higher BBTB permeability. Patients with SHH and Gp3 MB show heterogeneous contrast enhancement, suggesting that different parts of the same tumor have varying levels of BBTB permeability. Gp4 is characterized by small or non-enhancing tumors with a completely intact BBTB [78,79,80].

Another tumor that is thought to impact the permeability of the BBB is diffuse midline glioma (DMG), an aggressive pediatric brain tumor that develops diffusely in the brainstem [81,82]. Surgical treatment has limited utility due to its location, but chemotherapy and other targeted therapies are often not enough to improve survival [82,83]. The reason behind this inefficiency may be related to an intact BBTB [81,84,85]. In this context, the use of small lipophilic molecules that may cross the BBTB or MRI-guided focused ultrasound to create transient disruption of the BBTB has been proposed to increase the penetrance of drugs in the brain [65,86,87]. Taken together, medulloblastoma and diffuse midline glioma represent two examples of how variable the permeability of the BBB is in different types of lesions.

Further research is required to understand the precise role of the BBB in pHGG. Nonetheless, current research explores the possibility of bypassing the BBB to deliver antitumoral drugs, for example, by using nanotechnology to directly deliver drugs into the tumor [88]. For example, some recent studies have employed fucoidan-based nanoparticles that target P-selectin in activated endothelial cells. Radiotherapy can enhance P-selectin expression on endothelial cells, and, consequently, more nanoparticles can accumulate at the tumor site [89,90,91].

Another strategy to enhance drug delivery is to promote a transient opening of the BBB with MRI-guided focused ultrasound (MRgFUS). A study on murine DMG models by Martinez et al. showed that using MRgFUS with microbubbles can provide a temporary focal opening of the BBB, which allows for greater drug delivery to the tumor [92].

## 4. Immunotherapy and Targeted Therapies: New Therapeutic Strategies in pHGGs

Currently, high-grade glioma (HGG) therapies include chemoradiotherapy, surgery, targeted agents, and immunotherapy [89]. Despite the remarkable progress in surgical techniques and chemotherapy, HGGs remain a significant cause of pediatric brain tumors associated with a poor prognosis. Whenever possible, the initial treatment for pHGG should involve surgery, considering the tumor’s location and the patient’s clinical status. The aim is to obtain a maximally safe surgical resection, as gross-total resection (GTR) is a main factor in improving the prognosis [93]. However, despite GTR, total elimination of the cancer cells is often not possible due to the infiltrative nature of the disease [94,95]. Moreover, radiotherapy and chemotherapy have limited success and still present considerable adverse effects. Therefore, it is essential to consider and investigate other therapeutic strategies as additional or alternative treatments. For instance, target therapies and immunotherapy represent new promising strategies, having already shown some positive results in a few pHGG studies. Also, new combination strategies are under investigation, such as administering immunotherapies with radiotherapy [96]. Radiotherapy can have a modulative effect on the immune system and, thus, generate an immune response by enhancing tumor antigen exposure [97] and making the tumor more accessible to both innate and adaptive immune cells [98]. This response could create a positive synergistic effect if immunotherapy is simultaneously administered [99].

### 4.1. Methods to Study and Develop New Therapies

Accurate preclinical models of a disease are the key to developing new, tractable therapeutic strategies. The search for in vivo and in vitro models that can reliably assess drug efficacy and safety is crucial in the field of cancer research. For instance, cells immortalized with human telomerase ribonucleoprotein reverse transcriptase (hTERT) have been recently used as a method to generate DMG models. Such models appear tumorigenic in athymic rodents and faithfully recapitulate DMG’s infiltrative nature [100]. However, the main limitation of in vitro expanded and immortalized cell lines is the inevitable genetic and epigenetic drift that is driven by multiple rounds of mitotic division and by the external environment [101]. Studying high-grade gliomas in murine models can be achieved through three potential strategies. The first is the induction of a spontaneous tumor in the host by exposure to specific carcinogenic agents [102] or through viral transduction [103]. The advantage of spontaneously induced tumors is that they better mirror natural tumor evolution and microenvironmental changes. The second strategy is the creation of transgenic mouse systems focusing on the targeted genes and pathways involved in tumor progression [104]. The third possible model utilizes tumor cell implantation. This is achievable by realizing allograft models of mouse tumor cells for histone mutant glioma and inducing the expression of hallmark mutations [105]. Also, xenograft models from other species can be generated by successively injecting tumor cells into mice. For instance, creating a xenograft model of patient-derived glioblastoma is the best way to study the glioblastoma immune microenvironment [106]. The heterotopic transplantation of the tumor into the subcutaneous tissue is straightforward and useful for drug testing. However, it may negatively impact the tumor phenotype and impair the investigation of BBB penetration [107]. The choice of the mouse model for transplantation varies among different immunocompromised models but usually involves athymic nude mice [108]. Interestingly, adult immunocompetent mice fail to recapitulate the human-specific TME features, while embryonic (E12.5) mice can be adapted to achieve this feature [109]. The embryonic stage injection can therefore provide experimental glioblastoma that invades the mouse brain and exhibits the complex intact TME with vasculature, astrocytes, and immune cell infiltration.

In this section, we present some examples of models used to study pHGG. Due to the lack of studies focusing on pediatric hemispheric gliomas, we discuss some models that have been applied to recapitulate other pediatric HGGs. It is possible that some of them may be adapted to study pHGG.

To study the process of tumorigenesis, immune-competent mice can be genetically engineered to present with the most relevant mutation (genetically engineered mouse models, GEMMs). Yadavilli et al. created a GEMM model of DMG to induce *Ink4a-ARF* loss and “platelet-derived growth factor B” (*PDGFB)* overexpression using the replication-competent avian sarcoma-leucosis virus (RCAS) vector specifically targeting nestin-expressing cells in the pons [110]. More recently, another GEMM model was generated by DMG-induced tumor-specific genetic alterations, including *PDGF-B*, H3.3.K27M, and *p53*. In this case, DF1 (chicken fibroblast) cells have been engineered with RCAS-PDGFB, RCAS-Cre, and RCAS-H3.3K27M, thus creating DIPG-like tumors in the mouse brainstem [111]. Also, human embryonic stem-cell-derived neural progenitor cells (NPCs) have been used with an alternative approach to generate DMG in mice models. This model was created mimicking the characteristic PDGFRA-D842V mutation, inducing shRNA knockdown of *p53* and the H3.3K27M mutation through lentiviral modification [112]. Mohammad et al. generated DIPG-like tumors as characterized by H3K27 hypomethylation in addition to *Nestin*, *Olig2*, and *ATRX* expression [20]. Mouse neural stem cells were engineered to induce the expression of both the H3K27M mutation and *PDGF-B.* Consequently, with the transplantation of these cells into SCID mice pons, H3K27M and *p53* deletions were reproduced, thus replicating glioma formation. However, their result is still debated, and some authors, including Pathania et al., were unable to reproduce glioma formation [113].

McNicholas et al. [114] proposed two models to study pediatric HGGs and explore new therapeutic strategies. In their study, the main focus was the effect of different *PDGFR* mutations in cases with H3.3-G34R mutants and the responsiveness to new therapeutic strategies. Specifically, they developed a delivery system using in utero electroporation (IUE) of piggyBac transposons and CRISPR vectors to generate mutations on embryonic day 12.5 (E12.5) into the mouse dorsal or ventral pallium [115]. The clonal induction was consented to by the transient expression of the piggyBac transposase and Cas9. This stimulus was sufficient to create genetically recombined progenitors able to give rise to gliomas in an immunocompetent system [116]. In the same work, the authors confirmed the possibility of introducing site-specific genetic mutations at E12.5. To evaluate the contributions of specific PDGFRA alterations, McNicholas et al. [114] created three mouse models. The three models carry H3.3-G34R, *ATRX* loss of function, and *p53* loss of function (GPA), combined with *PDGFRA* wild-type (GPAP), *PDGFRA*-C235Y (GPAC), or *PDGFRA*-D842V (GPAD). The role of *PDGFRA* wild-type was to reproduce the focal amplification of the normal gene, which is frequently described in these tumors. Indeed, *PDGFRA*-C235Y mimics the most relevant mutation observed in H3.3-G34R tumors. Finally, a positive control, represented by *PDGFRA*-D842V, was necessary to compare the results with the other published models [14,18,117,118]. The effective presence of the introduced mutation was checked and validated in all models. The investigators then evaluated tumor latency and median overall survival (mOS). In the future, these models could be used to assess target therapy responsiveness. Moreover, McNicholas et al. [114] obtained cell lines of gliomaspheres (GS) from each model (GPAP, GPAD, and GPAC). As a proof of the reliability of the model, GS cells expressed H3.3-G34R and maintained *ATRX* and *p53* downregulation with *PDGFRA* overexpression, as expected. Thus, they reproduced the genetic background of the original model, and this model could be used to assess responses to new target therapies. Despite being less accurate than in vivo models, in vitro models, specifically GS, are a promising strategy when in vivo models are not viable.

A few models have been designed and validated for hemispheric pHGGs. However, in vitro and in vivo models used for similar pathological entities could also be used in this field of research, possibly including 3D models and organoids. Further investigations are necessary to develop and adapt models that accurately represent this tumor both in vitro and in vivo.

### 4.2. Targeted Therapies

Pediatric HGGs exhibit distinct molecular genetic profiles compared to their adult counterparts, requiring meticulous and focused research. Compared to adult HGG, pHGGs commonly harbor mutations in *PDGFRA* and *TP53*, along with K27M and G34R/V mutations on histone H3 [89]. Novel targeted therapies, including these targets, are under investigation by ongoing trials. In this section, we will summarize some of the potential targets for pHGG therapy.

#### 4.2.1. PDGFRA Mutation

*PDGFRA* mutation is particularly frequent in diffuse hemispheric glioma H3.3-G34-mutant subtype. In vitro assessments of *PDGFRA* inhibitors, such as Dasatinib, have shown promising results for this subtype [119].

Chen et al. [9] studied H3.3-G34-mutants and highlighted a novel essential mechanism for this subtype. Accordingly, they noticed that *PDGFRA* expression is promoted by an ectopic mechanism enhanced by the TF *GSX2*, finally leading to over-activation of this kinase. Cells acquiring *PDGFRA* increase activation, undergo clonal selection, and enhance MAPK signaling, thus promoting gliomagenesis. Nevertheless, these mechanisms have important therapeutic implications, as both *PDGFRA* mutations and MAPK activation are potentially targetable, even considering the choice of multiple targets in combination.

Preliminary findings suggest that combining *PDGFRA* inhibitors with *MET* inhibitors or mTOR inhibitors may enhance treatment efficacy [120]. The combination of Dasatinib with Everolimus was assessed in vivo and significantly improved overall survival. Another study assessed OS in recurrent pediatric gliomas with this combination in six cases with confirmed *PDGFRA*-driven gliomas. They achieved a higher median OS in comparison with the expected OS for recurrent pediatric gliomas [120]. Therefore, this combination warrants further investigation in forthcoming clinical trials.

McNicholas et al. [114] conducted an in vitro study with gliomaspheres to assess the sensitivity of H3.3-G34R tumors carrying different *PDGFRA* mutations (GPAP, GPAD, and GPAC GS cells, described in the previous paragraph) to eight targeted therapies, including inhibitors of PDGFRA (Avapritinib) and FGFR1 (Infigratinib) [121,122,123,124,125].

The findings from this group revealed the absence of a response of GPAC cells to the PDGFRA inhibitor (Avapritinib) [126]. Nevertheless, they first reported a noticeable sensitivity of these cells to the FGFR inhibitor (Infigratinib). Indeed, GPAD, GPAP, and wild-type NS did not report this level of sensitivity to Infigratinib. This result supports the unique selectivity of this drug for the *PDGFRA*-C235Y mutation.

In summary, the study demonstrated that GPAC cells are particularly sensitive to Infigratinib. Further research should validate these results and broaden the scope of evaluation to encompass tumors carrying different *PDGFRA* mutations.

#### 4.2.2. IDH Mutation

IDH mutations cause an accumulation of 2-hydroxyglutarate through interference with the Krebs cycle, thus promoting tumor progression [127]. This mutation is less frequent in pHGG in comparison with adult gliomas [11,128]. *IDH* mutations are more frequently found in older children [129,130] and are often associated with “methylguanine-DNA methyl-transferase” (MGMT) methylation, with the potential efficacy of temozolomide as a treatment [11,131].

Recent investigations have explored targeted therapies against IDH with TKIs such as Vorasidenib and Ivosidenib. However, current clinical trials with IDH inhibitors are focused on adults.

It was recently discovered that homologous recombination can be inhibited by 2-hydroxyglutarate, thus causing the missed repair of double-stranded breaks in IDH-mutated cells. This fact led to the hypothesis that PARP inhibitors could be an effective treatment strategy [127,132]. BRCA-mutant cancers have been effectively targeted by Olaparib, thereby b possibly applicable to pHGG patients with the *BRCA* mutation. Recently, patients treated with Olaparib and temozolomide (TMZ) have exhibited a positive response for over 2 years [133]. One current trial is investigating this combination (TMZ-Olaparib) with radiotherapy [134] and two ongoing clinical trials are evaluating Olaparib on pHGG patients (NCT03155620 [135] and NCT03233204 [136]). In the near future, *IDH*-mutant gliomas could be the focus of new combinatorial chemotherapeutic regimens, acting simultaneously on the *IDH* mutation itself and on its molecular consequences.

#### 4.2.3. BRAF Mutation

The inhibition of BRAF, being the first kinase of the MAPK pathway, acts by halting proliferation signaling. However, it must be noted that in *BRAF* wild-type cells, these drugs not only have poor activity, but they may also paradoxically activate the RAF-MEK-ERK pathway, thus augmenting the rate of proliferation. Accordingly, the assessment of the *BRAF* mutational state is necessary to decide whether to target this molecule or not. Consequently, BRAF inhibitors hold promise for the treatment of HGGs carrying the *BRAF*-V600E mutation. Some phase I and II studies showed promising outcomes with Vemurafenib [137], Selumetinib [138], and Trametinib [139,140,141] in low-grade gliomas. Unfortunately, the efficacy of this drug in HGG is less well understood. Nevertheless, the assessment of BRAF inhibitor monotherapy efficacy in *BRAF*-V600E mutant pHGG is under investigation (NCT03919071).

#### 4.2.4. NF1 Loss

The homozygous loss of *NF1* equally causes RAS constitutive activation and, as a consequence, MEK/ERK upregulation. In this situation, MEK inhibitors are potentially effective in suppressing the resulting enhanced proliferation. This strategy already showed positive results in LGGs and caused tumor regression in hemispheric pediatric HGGs [142]. NF1 tumors also exhibit increased immunological activity and are consequently more suitable for immunotherapy approaches [143].

#### 4.2.5. EGFR Amplification

*EGFR* or *Myc* amplifications are potentially targeted with Gefitinib, Erlotinib, and Afatinib. In adult gliomas, these therapies provided a well-tolerated treatment option but did not achieve successful efficacy levels. The combination of Gefitinib and radiotherapy was evaluated in pediatric patients but failed to demonstrate benefits in survival [144,145]. Recently developed EGFR inhibitors, such as Osimertinib, have shown good results in terms of preclinical efficacy, BBB penetration, and clinical outcomes (off-label therapy) [142,146]. In the case of pediatric patients, these new drugs should be further assessed and evaluated in combination with radiation therapy and traditional chemotherapies.

#### 4.2.6. HDAC Inhibition

Evidence indicates that histone mutations cause impacting epigenetic changes, including both the silencing and the activation of genes. For this reason, strategies aimed at interrupting the occurrence of these mutations should equally have an impact on tumorigenesis. Several ongoing trials are investigating “histone deacetylase” (HDAC) inhibitors in DIPG. The first reported results include a phase I trial assessing the combination of an HDAC inhibitor (Panobinostat) and radiotherapy on H3K27M DMG. To date, HDAC inhibition in H3.3-G34R/V mutants has not demonstrated encouraging results [147]. Nevertheless, further elucidation and evaluation of these drugs’ mechanisms and potential off-target effects are necessary [148].

#### 4.2.7. AURKA Inhibition

H3.3-G34V mutants have been reported to induce an augmented expression of *MYCN*, thus increasing proliferative activity [16]. MYCN can be targeted by inhibiting its stabilizers, such as “checkpoint kinase 1” (Chk1) and AURKA kinase. Concerning the inhibition of AURKA, in vitro studies demonstrated the selectivity of the inhibitor (VX-689) against H3.3-G34V mutant cells [16].

Pediatric HGG encompasses a group of heterogeneous entities characterized by multiple molecular mutations and features, with implications for diagnostics, clinical characterization, and treatment optimization. Future clinical trials on pHGG should focus on identifying relevant biomarkers and stratifying patients into subgroups based on their molecular features. Moreover, combination therapies may provide further clinical benefits, but they require additional systematic investigation.

### 4.3. Immune System Considerations

Pediatric gliomas present two distinct immune microenvironmental phenotypes: they are either characterized as immunogenic (hot) or immunosuppressive (cold). Studies have shown that hot pHGGs express high levels of immune checkpoint molecules (ICMs) and are more responsive to anti-PD1 and anti-CTLA4 treatments. Conversely, cold HGGs display minimal expression of ICM and are unlikely to benefit from ICM-targeted treatments [149].

Among pediatric gliomas, DMGs are distinguished by a microenvironment that is neither highly immunosuppressive nor inflammatory. This is attributed to the scarcity of antigen-presenting cells in the tumor environment and the low mutational burden of DMG, which together hinder immunosurveillance against tumorigenesis and progression [150]. Consequently, this tumor represents a prospectively poor responder to immune checkpoint inhibition. For this reason, DMG represents the perfect candidate for cellular immunotherapies, including chimeric antigen receptor (CAR) T cell therapies or ex vivo expanded natural killer (NK) cell therapies.

H3.3-G34R mutants appear to be more susceptible to immunotherapies. This specific histone mutation appears to modify the regulatory elements within the JAK/STAT pathway, resulting in hyperactivation. As a result, these tumors undergo changes in their immune TME, favoring an immune-permissive phenotype. This rationale explains the expected susceptibility of these tumors to immune-stimulatory gene therapy, such as immunostimulatory therapy with TK/Flt3L [21].

### 4.4. Immunotherapeutic Strategies

Immunotherapeutic modalities include immune checkpoint inhibitors (ICIs), antibody-mediated immunotherapy, cancer vaccines, adoptive cellular therapy, and oncolytic viral therapy [105]. Despite the lack of studies focusing on immunotherapeutic approaches to target hemispheric pHGG, several lines of research are evaluating immunotherapy for H3.3 K27M DMGs. The main efforts focus on the effects of targeting neoantigens generated by histone mutations through T-cell therapy or targeted vaccines [151], especially for H3.3 K27M tumors. Moreover, approaches not focused on targeting the oncohistone mutation are ongoing with promising results, with studies involving hemispheric HGGs [147]. In Table 1, we summarize the current immunotherapeutic clinical trials for pediatric CNS tumors (last updated, April 2024). It is of relevance that out of 49 clinical trials, 40 included H3G34 hemispheric glioma in the broad category of pediatric brain tumors, and only one had a specific arm of the clinical trial focused on hemispheric gliomas (NCT05298995). Given the paucity of studies centered on pediatric hemispheric gliomas and the peculiarity of the genetic background of these tumors, we highlight the need for more basket clinical trials focused on specific targeting of the H3 G34 mutation and also studies specifically focusing on Diffuse Hemispheric Glioma, H3 G34-Mutants.

#### 4.4.1. Immune Checkpoint Inhibitors (ICIs)

Immune checkpoint inhibitors (ICIs) were first generated and assessed against adult tumors, providing promising results [159,160]. In comparison, these therapies have not been equally considered for pediatric tumors. ICIs, including Ipilimumab, Nivolumab, and Pembrolizumab, have been used off-label in 11 pediatric patients with recurrent brain tumors [161]. The study reported good tolerability of these drugs, but it is difficult to evaluate their efficacy considering the limited number of patients included. An ongoing phase 1 clinical trial has the aim of defining the optimal dose and the possible adverse events for Pembrolizumab in pHGG (clinical trial: NCT02359565).

Regarding ICIs, some concerns must be considered, such as tumor mutational burden, expression of PD-L1 by tumor cells and TME, and infiltration of PD-1+ T cells. Majzner et al. proved that PD-L1 expression is relevant only in 36% of pHGG (5 of 14 samples) [162]. However, the limited size of the sample makes it difficult to generalize this result to HGGs. Moreover, pHGG frequently presents with a low tumor mutational burden, thus possibly interfering with ICI effectiveness [163]. The expression of PDL1 and its correlation with tumor mutational burden must be further evaluated in pHGGs. Some authors suggest that the possible use of ICIs in pHGG should be explored as an adjuvant to other strategies, such as cancer vaccines and CAR-T cells [164].

#### 4.4.2. Antibody-Mediated Immunotherapy

The use of antibodies targeting immunosuppressive molecules in cancer is a promising strategy deserving further assessment for pediatric gliomas. CD47 is defined as a “don’t eat me” signal molecule that inhibits phagocytosis by binding to receptors in macrophages and microglia. The monoclonal antibody anti-CD47 (Hu5F9-G4) was studied in preclinical in vivo models, resulting in increased survival and a reduction in tumor volume in pHGG [165]. Clinical trials for anti-CD47 determined its tolerability and efficacy in adult tumors [166] but did not address the same issue in pediatrics.

CD40 is a molecule commonly expressed in APCs [167]. In other cancers, the binding of CD40 to its ligand (CD154/CD40L) triggers an inflammatory and antitumor response [167]. Also, an anti-CD40 antibody was tested using in vivo models of glioma and was able to augment the antitumor activity of immune cells [168,169]. In pHGG, the anti-CD40 (APX005M) is under evaluation for its efficacy (NCT03389802).

#### 4.4.3. Cancer Vaccines

Cancer vaccines deliver tumor antigens to be loaded into MHCs of Antigen Presenting Cells (APCs) for the purpose of activating tumor-specific T cells. Preclinical and clinical studies have provided encouraging results despite being frequently limited to specific HLA haplotypes. This characteristic narrows the number of treatable patients. Future studies should extend to patients harboring different HLA haplotypes and to the use of peptide vaccines.

#### 4.4.4. Adoptive T-Cells and Dendritic Cells

A novel approach to leveraging immune cells to target antigens on the surface of tumor cells is represented by Adoptive T-cell therapies. In particular, T cells transduced with H3.3K27M-specific TCR have been studied [170]. Currently, there are no studies evaluating Adoptive T-cell therapies in hemispheric pHGG.

A different approach is the use of autologous dendritic cells (DCs). DCs are isolated from the patient and loaded with tumor antigens, which are isolated from cell lysates or tumor cDNA libraries. According to the few currently concluded trials, pediatric glioma patients seem to be good responders to DC immunotherapy [171,172]. For this reason, trials with DC vaccination in this patient subgroup must be encouraged [173]. 

#### 4.4.5. CAR-T Cells

CAR-T cells have been designed to target specific tumor antigens. Differently from other therapeutic strategies, CAR-T does not act on the endogenous immune response directly, but it downregulates tumor antigen expression. An ongoing phase 1 clinical trial is evaluating anti-IL13aR2 CAR-T cells in recurrent or relapsed gliomas (NCT02208362), enrolling patients between the ages of 12 and 75 years.

Considering pediatric gliomas, this strategy has almost exclusively been studied in diffuse midline gliomas (DMGs). Against these tumors, preclinical studies have provided promising results targeting the mutant K27M [151,174] and the ganglioside GD2 [175]. In fact, the targeting of GD2 has demonstrated effective inhibitory effects on tumorigenesis [175]. Despite the side effects of neuro-inflammation and edema, the preliminary results from a CAR-T cell ongoing clinical trial appear promising from clinical and radiological evaluation in the four enrolled patients included in the trial [176]. CAR-T cells potential targets and role in hemispheric pHGG require further investigation.

#### 4.4.6. IDO Inhibitors

Glioma cells use various immunosuppressive mechanisms. One of these strategies is the overexpression of “Indoleamine 2,3-dioxygenase” (IDO) [177], an enzyme producing kynurenines from tryptophan. This mechanism reduces the availability of tryptophan for DC maturation and T-cell survival. Considering this mechanism, preclinical in vivo models to assess the inhibition of IDO have been generated [177]. A phase 1 clinical trial evaluated the efficacy of an IDO inhibitor (Indoximod) in pHGG (NCT02502708). The study has been completed and demonstrated that Indoximod could be safely combined with other adjuvant treatments. Considering these results, future Phase II/III trials for pediatric brain tumors could be planned [178].

#### 4.4.7. Targeting the Immune Microenvironment

Some studies have focused on the interaction between tumor cells and the surrounding microenvironment, promoting tumor growth [105,179,180]. Enhancer of “zeste homolog 2” (EZH2) is the enzymatic component of the “polycomb repressor complex 2” (PCR2), likely playing a crucial role in the development of H3k27M DMG. The H3K27M mutation seems to oppose the function of EZH2, which would otherwise act as a tumor suppressor [181]. As a result, this leads to an abnormal methylation pattern, leading to an abnormal transcriptome that characterizes this tumor [182]. This led to great interest in developing therapeutic strategies targeting this enzyme [183]. Studies focused on targeting EZH2 directly in DMG cells had poor overall results in vitro [20]. However, inhibiting EZH2 in vivo was promising, leading to several studies focusing on the role of the tumor microenvironment in contributing to the tumor suppressive effect of EZH2 inhibition. In H3.3-K27M patient-derived pHGG cell lines, the inhibition of EZH2 in microglia is able to decrease tumor growth and migration. This finding is a possible indicator of the crosstalk present within the tumoral microenvironment that supports tumor growth [182]. In the context of tumors, EZH2 is a target for immunotherapeutic strategies aimed at modulating the TME. Such strategies could pave the way for many other approaches focusing on the immune microenvironment as an appealing means of indirectly targeting the tumor. Similar strategies for investigating the TME impact on shaping tumor treatment responses should also be evaluated for hemispheric pHGG. 

### 4.5. Convection-Enhanced Delivery (CED)

CED utilizes the implantation of a catheter to infuse drugs directly into the tumor itself, thus bypassing the BBB. Through a pressure gradient generated by the pump, the volume of distribution is more homogeneous [184]. This results in higher drug concentrations in the target area and reduced systemic involvement. This technique could enhance the delivery of drugs with poor BBB penetration or systemic toxicity [185,186,187,188].

The majority of studies with CED have involved adult HGG, frequently recruited after the canonical adjuvant treatment [189,190]. A few studies evaluated monoclonal antibody delivery via CED, but this field should be further explored. Considering pediatric tumors, the recent use of CED has been mainly focused on DIPG [191]. This approach may offer an interesting perspective for delivering promising new pHGG therapies that cannot be administered systemically, such as CRISPR-Cas constructs.

### 4.6. Immunotherapy and Corticosteroids

Corticosteroids are widely used in patients with pHGG to ameliorate tumor edema-related symptoms. However, corticosteroids can negatively impact the response to immunotherapy by decreasing the migration of leukocytes, reducing blood counts of leukocytes, especially concerning the subset of T cells, and decreasing the level of IgG produced. The PNOC-007 trial of the H3.3K27M peptide vaccine in children with DMG [94] provided insight on the inhibitory effects of steroid therapy on vaccine-specific T-cell responses. In this study, patients treated with dexamethasone presented a decreased expansion of CD8+ T cells reactive to the treatment [192]. ICIs may have an antagonistic relationship with corticosteroids, even though this issue has not been extensively explored yet [193].

### 4.7. Focus on H3.3-G34 Mutants

Therapeutic approaches to target H3.3-G34 mutant gliomas have been poorly investigated compared to H3 K27M DMG. However, H3.3-G34R glioma cells have been analyzed, showing epigenetic dysregulation that enhances autocrine/paracrine stimulation of STAT3, thus stimulating tumor growth [98].

Moreover, a screening study of H3.3-K27, H3.3-G34R, and H3.3-WT cell lines highlighted that the different oncohistone mutations are not inferring unique vulnerabilities. Drug response variability to immunotherapies was associated with intertumoral heterogeneity, as with oncohistones [73]. Bressan et al. [14] studied the role of G34R mutations, along with mutations in other critical oncogenes, using in vitro and orthotopic in vivo experiments. The mutation was introduced in neural cells from different regions of the brain, including the hindbrain and the forebrain. Especially in the latter location, the mutation induced oncogenesis. However, the G34R mutation was not extremely oncogenic per se. In fact, it required additional mutations, such as *PDGFRA* amplification and *P53* loss, to promote tumor development. Moreover, the G34R mutation played a role in alternative splicing and modulation of Notch signaling. When Notch2l was overexpressed, it caused an increase in the proliferation of progenitor cells in the forebrain but not in the hindbrain. This could potentially explain why the effects of G34R alteration differ depending on location [18]. Also, it may also explain why there are regional differences in the occurrence of the two most relevant histone 3 mutations (G34R/V and K27M). 

Alongside targeted studies and treatment strategies, preclinical studies should focus on identifying synergistic therapeutic combinations and consider the locoregional impact of each genetic abnormality on tumorigenesis [147]. This could help us better understand neurogenesis in the developing brain and inform a more tailored design of treatment modalities. Finally, brain location appears to be a factor contributing both to driving and shaping the oncogenic potential.

### 4.8. Combination with Temozolomide

Despite the interesting and expanding scenario of therapies concerning pHGGs, treatment effectiveness has not provided impactful results as yet. Chemotherapy, mostly represented by temozolomide, reported poor results in pHGG in comparison with adult gliomas [18]. Phase I and II trials have been carried out concerning the use of temozolomide in pHGG, but the most effective schedule of administration still needs to be defined [194]. Thus, the future of temozolomide for pediatric gliomas may concern combinatory use with other drugs, so that the effect may be boosted and the side effects, such as lymphopenia, could be limited. Moreover, some studies have shown that following treatment with temozolomide, the tumor mutational load can increase [152]. This increase is related to a higher amount of neoantigens, potentially increasing the intensity and duration of the response to immunotherapy. Considering this interesting perspective, the effectiveness of immunotherapy may be enhanced, providing more impactful results than immunotherapy alone. Nevertheless, few trials have investigated the effects of temozolomide with immunotherapy [133,134].

### 4.9. Limitations and Future Perspectives

The development and introduction of immunotherapies for pHGG has been a slow progression compared to adult gliomas. Further research is required to comprehend the complex interaction between the tumor and the immune system, especially concerning how this interaction in each pHGG subtype is established and maintained. Moreover, most pHGG molecular subtypes still cannot be extensively investigated because of the lack of a murine model. This issue is currently preventing accurate preclinical evaluation of the available immunotherapies. Additionally, combination therapies require further evaluation.

The question is even more complicated if we consider that genomic and transcriptomic profiles are not directly determinants of the prediction of response to a particular treatment. Precision therapies may have different responses in patients presenting similar mutations. In fact, the combination of genetic and epigenetic alterations is unique for each patient, and the sensitivity to a particular treatment derives from this complex landscape. For this reason, personalized treatments could result in the best clinical responses.

Currently, the assessment of individual responses to available treatments can be performed by collecting patient-derived tumor cells, expanding them ex vivo or in vitro, and confirming their response. This can be time-consuming, thus preventing cases of advanced pHGGs from benefiting from the findings. In vitro systems can be completed in two weeks, but results are less predictive of human responses. Unfortunately, the technological equipment that is necessary to evaluate single-cell heterogeneity (such as RNA-seq) is not frequently available in clinical daily practice. These approaches could be further developed in the future, thus allowing for more personalized and subtype-specific therapies.

## 5. Conclusions

The complex biological, genomic, and epigenomic background of pHGG has proven difficult with regard to developing safe and efficacious treatment options. The role of the microenvironment, the functionality of the BBB, and the use of convection-enhanced delivery in these tumors should be explored for their potential roles in treatment efficacy. Promising actionable targets have been identified, and the response to these targeted treatments needs to be further evaluated. The more appealing targets for H3.3-G34 mutant gliomas are represented by PDGFRA, BRAF, and IDH1. Moreover, studies concerning the complex interaction between the tumor and the immune system should be moved forward to establish the potential role of immunotherapy for pHGG. Finally, we propose that new emerging therapeutic strategies should be explored in pHGGs.

## Figures and Tables

**Table 1:** Immunotherapeutic clinical trials targeting pediatric CNS tumors (current as of April 2024), source: ClinicalTrials.Gov. Trials have been divided considering the therapeutic strategy and pointing out NCT number, focus of the study, study status, conditions included, interventions performed. Additionally, the specific inclusion of H3.3-G34 mutants has been considered. For each immunotherapeutic strategy, a previous table includes the definition and pros/cons of its application.

CAR T					
DEFINITION		PROS		CONS	
Chimeric antigen receptor T cells. Composed by an antigen binding domain, a transmembrane portion and an intracellular part. T cells of the patient are collected and engineered via a viral vector t express a receptor for an antigen of interest. Cells are then expanded and infused [152].		– high efficacy in some cancer types – Specific antigenic target – prolonged response duration – applicability to several cancer types – deactivation strategies		– side effects (cytokine release syndrome and neurotoxicity) – costs – complexity of manufacturing process – limited efficacy in solid tumors – cancer resistance – need for expertise and dedicated structures	
NCT Number	Focus of the Study	Study Status	Conditions	Interventions	H3G34 inclusion
NCT05768880	CAR T Cell Locoregional Immunotherapy targeting B7-H3, EGFR806, HER2, And IL13-Zetakine	RECRUITING	DIPG, DMG, Recurrent CNS Tumor, Refractory Primary Malignant CNS tumor	SC-CAR4BRAIN	Y (non-specific)
NCT05298995	GD2-CAR T Cells	RECRUITING	Pediatric Brain Tumor, Medulloblastoma, Embryonal Tumor, HGG, DMG, DIPG, Brain Tumor Adult	GD2-CART01 (iC9-GD2-CAR T-cells)	Y (specific arm for H3G34)
NCT04510051	CAR T Cells for IL13Rα2 positive tumors	RECRUITING	Malignant Brain Neoplasm, Recurrent Malignant Brain Neoplasm, Refractory Malignant Brain Neoplasm	Cyclophosphamide, Fludarabine, IL13Ralpha2-specific Hinge-optimized 41BB-co-stimulatory CAR Truncated CD19-expressing Autologous T-Lymphocytes	Y (non-specific)
NCT04483778	B7H3 CAR T Cell Immunotherapy	ACTIVE_NOT_RECRUITING	Pediatric Solid Tumor, Germ Cell Tumor, Retinoblastoma, Hepatoblastoma, Wilms Tumor, Rhabdoid Tumor, Carcinoma, Osteosarcoma, Ewing Sarcoma, Rhabdomyosarcoma, Synovial Sarcoma, Clear Cell Sarcoma, Malignant Peripheral Nerve Sheath Tumors, desmoplastic Small Round Cell Tumor, Soft Tissue Sarcoma, Neuroblastoma, Melanoma	Second generation 4-1BBζ B7H3-EGFRt-DHFR, second generation 4-1BBζ B7H3-EGFRt-DHFR (selected) and a second generation 4-1BBζ CD19-Her2tG, Pembrolizumab	N
NCT04185038	B7-H3-Specific CAR T Cell Locoregional Immunotherapy	RECRUITING	Central Nervous System Tumor, DIPG, DMG, Ependymoma, Medulloblastoma, Childhood, Germ Cell Tumor, Atypical Teratoid/Rhabdoid Tumor, Primitive Neuroectodermal Tumor, Choroid Plexus Carcinoma, Pineoblastoma, Childhood, Glioma	SCRI-CARB7H3(s), B7H3-specific chimeric antigen receptor (CAR) T cell	Y (non-specific)
NCT04099797	C7R-GD2.CAR T Cells for GD2-expressing Brain Tumors (GAIL-B)	RECRUITING	DIPG, HGG, Embryonal Tumor, Ependymal Tumor	C7R-GD2.CART cells, C7R-GD2.CART cells	Y (non-specific)
NCT03638167	EGFR806-specific CAR T Cell Locoregional Immunotherapy in EGFR-positive tumors	ACTIVE_NOT_RECRUITING	Pediatric CNS tumors	EGFR806-specific chimeric antigen receptor (CAR) T cell	Y (non-specific)
NCT03618381	EGFR806 CAR T Cell Immunotherapy	RECRUITING	Pediatric Solid Tumor	Second generation 4-1BBζ EGFR806-EGFRt, second generation 4-1BBζ EGFR806-EGFRt and a second generation 4 1BBζ CD19-Her2tG	Y (non-specific)
NCT03500991	HER2-specific CAR T Cell Locoregional Immunotherapy for HER2-positive tumors	ACTIVE_NOT_RECRUITING	Pediatric CNS tumors, Glioma, Ependymoma, Medulloblastoma, Germ Cell Tumor, Atypical Teratoid/Rhabdoid Tumor, Primitive Neuroectodermal Tumor, Choroid Plexus Carcinoma, Pineoblastoma	HER2-specific chimeric antigen receptor (CAR) T cell	Y (non-specific)
NCT02442297	T Cells Expressing HER2-specific Chimeric Antigen Receptors(CAR)	ACTIVE_NOT_RECRUITING	Brain Tumor, Recurrent, Brain Tumor, Refractory	HER2-specific T cells	Y (non-specific)
PEPTIDE VACCINE					
DEFINITION		PROS		CONS	
Vaccine composed by a peptidic tumor antigen, adjuvants and a delivery system. It is aimed at eliciting an immune response against a specific antigen [153].		– safety – specificity – ease of conservation – ease of target modification – broad applicability		– low immunogenicity – need for boosters – HLA dependency – tumor heterogeneity and immune evasion – limited activity in immunocompromised – need for adjuvants	
NCT Number	Focus of the Study	Study Status	Conditions	Interventions	H3G34 inclusion
NCT04808245	Peptide vaccine	RECRUITING	Newly Diagnosed H3-mutated Glioma	Tecentriq, H3K27M peptide vaccine, Imiquimod	N
NCT04749641	Neoantigen Vaccine Therapy	RECRUITING	DIPG	Histone H3.3-K27M Neoantigen Vaccine Therapy	N
NCT04573140	RNA-lipid Particle (RNA-LP) Vaccines	RECRUITING	Adult Glioblastoma	Autologous total tumor mRNA and pp65 full length (fl) lysosomal associated membrane protein (LAMP) mRNA loaded DOTAP liposome vaccine	Y (non-specific)
NCT02358187	A Vaccine Trial	RECRUITING	Low Grade Glioma	HLA-A2 Restricted Glioma Antigen-Peptides with Poly ICLC	N
NCT02960230	H3.3K27M Peptide Vaccine + Nivolumab	COMPLETED	DIPG, DMG, H3 K27M-Mutant	K27M peptide, Nivolumab	N
NCT01130077	Glioma Associated Antigen Vaccines in Conjunction With Poly ICLC	ACTIVE_NOT_RECRUITING	Newly Diagnosed Pediatric Pontine Glioma|Newly Diagnosed Pediatric HGG, Recurrent Pediatric High Grade Glioma, Recurrent Pediatric Low Grade Glioma	HLA-A2 restricted glioma antigen peptides vaccine, Poly ICLC	Y (non-specific)
NCT04943848	rHSC-DIPGVax + Checkpoint Blockade	RECRUITING	DIPG, DMG,, H3 K27M-Mutant	rHSC-DIPGVax, Balstilimab, Zalifrelimab	N
NCT03299309	PEP-CMV	ACTIVE_NOT_RECRUITING	Recurrent Medulloblastoma, Recurrent Brain Tumor, Childhood, Malignant Glioma	PEP-CMV	Y (non-specific)
NCT05096481	PEP-CMV Vaccine Targeting CMV Antigen	NOT_YET_RECRUITING	HGG, DIPG, Recurrent Medulloblastoma	PEP-CMV, Temozolomide, Tetanus Diphtheria Vaccine	Y (non-specific)
DENDRITIC CELLS					
DEFINITION		PROS		CONS	
Dendritic cells are antigen presenting cells responsible for the presentation of antigens to T cells and the regulation of the immune response. Cells are collected, exposed to a specific tumor antigen and infused, to elicit an immune response [154].		– strong and durable immune activation – personalized target – possibility to target many antigens at the same time		– production costs and complexity – tumor immune escape – individual variability of the response – autoimmunity – exaggerated inflammatory response	
NCT Number	Focus of the Study	Study Status	Conditions	Interventions	H3G34 inclusion
NCT03396575	Adoptive Cellular Therapy in Focal Radiotherapy Recovery with without temozolomide	ACTIVE_NOT_RECRUITING	DIPG, Brain Stem Glioma	TTRNA-DC vaccines with GM-CSF, TTRNA-xALT, Cyclophosphamide + Fludarabine Lymphodepletive Conditioning, Dose-Intensified TMZ, Td vaccine, Autologous Hematopoietic Stem Cells (HSC)	N
NCT03334305	Adoptive Cellular Therapy	ACTIVE_NOT_RECRUITING	Malignant Glioma, High Grade Glioma	TTRNA-DC vaccines with GM-CSF, Dose-intensified TMZ, Autologous Hematopoietic Stem cells (HSCs), TTRNA-xALT, Td vaccine	Y (non-specific)
NCT00107185	Vaccine Therapy	COMPLETED	Brain and Central Nervous System Tumors	Therapeutic autologous dendritic cells	Y (non-specific)
NCT03615404	Cytomegalovirus (CMV) RNA-Pulsed Dendritic Cells	COMPLETED	Pediatric Brain Tumor	CMV-DCs with GM-CSF|BIOLOGICAL: Td (tetanus toxoid)	Y (non-specific)
NCT04911621	Adjuvant Dendritic Cell Immunotherapy	ACTIVE_NOT_RECRUITING	HGG, DIPG	Dendritic cell vaccination + temozolomide-based chemoradiation Dendritic cell vaccination +/− conventional next-line treatment	Y (non-specific)
ONCOLYTIC VIRUSES					
DEFINITION		PROS		CONS	
genetically engineered or natural viruses to infect and kill cancer cells and to activate an immune response [155].		– tumor cell specificity – action not limited to primary tumor location – dual oncolytic and immune activation action – long lasting action		– low immunogenicity – need for boosters HLA dependency – tumor heterogeneity and immune evasion – limited activity in immunocompromised – need for adjuvants	
NCT Number	Focus of the Study	Study Status	Conditions	Interventions	H3G34 inclusion
NCT04482933	HSV G207 + one-dose radiation	NOT_YET_RECRUITING	Neoplasms, HGG, Glioblastoma Multiforme, Malignant Glioma of Brain, Anaplastic Astrocytoma of Brain, Anaplastic Glioma, Giant Cell Glioblastoma	Biological G207	Y (non-specific)
NCT03911388	HSV G207	ACTIVE_NOT_RECRUITING	Brain tumors	G207	Y (non-specific)
NCT03043391	PVSRIPO	COMPLETED	Malignant Glioma, Anaplastic Astrocytoma, Anaplastic Oligoastrocytoma, Anaplastic Oligodendroglioma, Glioblastoma, Gliosarcoma, Atypical Teratoid/Rhabdoid Tumor of Brain, Medulloblastoma, Ependymoma, Pleomorphic Xanthoastrocytoma of Brain, Embryonal Tumor of Brain	Polio/Rhinovirus Recombinant (PVSRIPO)	Y (non-specific)
NCT02457845	HSV G207 with or without a Single Radiation Dose	COMPLETED	Supratentorial Neoplasms, Malignant, Malignant Glioma, Glioblastoma, Anaplastic Astrocytoma, PNET, Cerebral Primitive Neuroectodermal Tumor, Embryonal Tumor	G207	Y (non-specific)
NCT02962167	Modified Measles Virus (MV-NIS)	COMPLETED	Medulloblastoma, Childhood, Recurrent, Atypical Teratoid/Rhabdoid Tumor, Medulloblastoma Recurrent	Modified Measles Virus,	N
NATURAL KILLER CELLS					
DEFINITION		PROS		CONS	
Immunotherapy based on the use of NK cytotoxic lymphocytes, autologous, allogeneic or engineered to express a chimeric antigen receptor [156].		– rapid and broad range activity – overcome immune evasion by MHC I downregulation – reduced risk of graft versus host disease – potential synergism with other immunotherapy strategies – potential of specific target by engineering – memory-like processes		– limited persistence and expansion – immune evasive tumor microenvironment – variable responses in different patients – cost and challenges of production – tumor evasion	
NCT Number	Focus of the Study	Study Status	Conditions	Interventions	H3G34 inclusion
NCT01875601	NK White Blood Cells and Interleukin	COMPLETED	Solid Tumors, Brain Tumors, Sarcoma, Pediatric Cancers, Neuroblastoma	Recombinant human interleukin-15 (rhIL-15), NK Cell Infusion	Y (non-specific)
NCT04730349	Bempegaldesleukin (BEMPEG: NKTR-214) + Nivolumab	TERMINATED	Ependymoma, Ewing Sarcoma, HGG, Leukemia and Lymphoma, Medulloblastoma, Miscellaneous Brain Tumors and Solid Tumors, Neuroblastoma, Relapsed, Refractory Malignant Neoplasms, Rhabdomyosarcoma	Nivolumab, NKTR-214	Y (non-specific)
NCT02100891	Haploidentical Transplant and Donor Natural Killer Cells for Solid Tumors	COMPLETED	Ewing Sarcoma, Neuroblastoma, Rhabdomyosarcoma, Osteosarcoma, CNS Tumors	Allogeneic HCT, Donor NK Cell Infusion	Y (non-specific)
ADENOVIRUS GENE THERAPY					
DEFINITION		PROS		CONS	
Immunotherapy based on the use adenovirus vector to deliver genes to tumor cells. The aim of the genes delivered can be the suppression tumor growth, the induction of cancer cell death and the initiation of anti-tumor immune response [157].		– effective delivery and high expression of target gene – highly versatile – potential synergism with other immunotherapy strategies – immediate activity		– rapid viral clearance if pre-existing immunity – inflammatory response – short term expression – toxicity and off target effects – heterogeneity in tumor expression of target can impair the efficacy and promote resistance	
NCT Number	Focus of the Study	Study Status	Conditions	Interventions	H3G34 inclusion
NCT03330197	Ad-RTS-hIL-12 + Veledimex i	TERMINATED	Pediatric Brain Tumor, DIPG	Ad-RTS-hIL-12|DRUG: Oral Veledimex—Arm 1 (Pediatric Brain Tumor), Oral Veledimex—Arm 2 (DIPG)	Y (non-specific)
NCT00634231	AdV-tk + Prodrug Therapy + Radiation Therapy	COMPLETED	Malignant Glioma|Recurrent Ependymoma	AdV-tk, valacyclovir, Radiation	Y (non-specific)
IMMUNE CHECKPOINT INHIBITORS					
DEFINITION		PROS		CONS	
Immunotherapeutic strategy aiming at blocking immune checkpoints, in order to reactive immune response against the tumor [158].		– – broad applicability – specific targeting – durable response		– immune-related adverse events – variable response rates – cost – delayed onset of action	
NCT Number	Focus of the Study	Study Status	Conditions	Interventions	H3G34 inclusion
NCT05106296	Chemo-immunotherapy: Ibrutinib + Indoximod	RECRUITING	Ependymoma, Medulloblastoma, Glioblastoma, Primary Brain Tumor	Ibrutinib, Indoximod, Cyclophosphamide, Etoposide	Y (non-specific)
NCT04323046	Neoadjuvant vs. adjuvant Immunotherapy	RECRUITING	Glioblastoma, Malignant Glioma, Recurrent Glioblastoma, Recurrent Malignant Glioma, Recurrent Grade III Glioma, Grade III Glioma	Nivolumab, Quality-of-Life Assessment	Y (non-specific)
NCT03690869	REGN2810 and REGN2810 in + Radiotherapy	TERMINATED	Relapsed/refractory Solid Tumor, Relapsed/refractory Central Nervous System Tumor, DIPG	Cemiplimab, radiation therapy	Y (non-specific)
NCT03130959	Nivolumab Monotherapy and Combination With Ipilimumab	COMPLETED	Various Advanced Cancers	Nivolumab, Ipilimumab	Y (non-specific)
NCT03451825	Avelumab	COMPLETED	Refractory or Relapsed Solid Tumors, Lymphoma	Avelumab	Y (non-specific)
NCT02793466	Durvalumab	COMPLETED	Solid Tumor, Lymphoma, CNS tumors	Durvalumab; MEDI4736	
NCT02992964	Pilot Study of Nivolumab in Pediatric Patients With Hypermutant Cancers	TERMINATED	Refractory or Recurrent Hypermutated Malignancies|Biallelic Mismatch Repair Deficiency (bMMRD) Positive Patients	Nivolumab	Y (non-specific)
NCT02359565	Pembrolizumab	RECRUITING	Constitutional Mismatch Repair Deficiency Syndrome, Lynch Syndrome, Malignant Glioma, Recurrent Brain Neoplasm, Recurrent Childhood Ependymoma|Recurrent DIPG, Recurrent Medulloblastoma, Refractory Brain Neoplasm, Refractory DIPG, Refractory Ependymoma, Refractory Medulloblastoma	Pembrolizumab, multiple imaging modalities	Y (non-specific)
MIXED IMMUNOTHERAPY STRATEGIES					
NCT Number	Focus of the Study	Study Status	Conditions	Interventions	H3G34 inclusion
NCT04049669	Indoximod + chemotherapy and radiation	RECRUITING	Glioblastoma, Medulloblastoma, Ependymoma, DIPG	Indoximod, Partial Radiation, Full-dose Radiation, Temozolomide, Cyclophosphamide, Etoposide, Lomustine	Y (non-specific)
NCT04408092	GM-CSF effect on Macrophages	COMPLETED	Ependymoma	Granulocyte Macrophage Colony Stimulation Factor	*N*
NCT06193759	Adoptive Cellular Therapy	NOT_YET_RECRUITING	Brain Tumor	Cytotoxic T lymphocytes (TSA-T) directed against proteogenomically determined multi-tumor specific antigens	Y (non-specific)
NCT03652545	Multi-antigen T Cell Infusion	RECRUITING	Brain Tumor	TAA-T	Y (non-specific)
NCT03389802	APX005M	ACTIVE_NOT_RECRUITING	Glioblastoma Multiforme, High-grade Astrocytoma NOS, CNS Primary Tumor, Nos, Ependymoma, NOS, DIPG, Medulloblastoma	APX005M treatment for recurrent or refractory primary malignant CNS tumor patients APX005M treatment for newly diagnosed DIPG patients	Y (non-specific)
NCT02813135	Therapeutic Stratification Trial of Molecular Anomalies in Relapsed or Refractory Tumors	RECRUITING	Pediatric Cancer	Ribociclib, Topotecan, Temozolomide, Everolimus, Adavosertib, Carboplatin, Olaparib, Irinotecan, Vistusertib, Nivolumab, Cyclophosphamide: Selumetinib, Enasidenib, Lirilumab, Fadraciclib, Cytarabine, Dexamethasone, Ceralasertib, Futibatinib, Capmatinib	Y (non-specific)
OTHER					
NCT Number	Focus of the Study	Study Status	Conditions	Interventions	H3G34 inclusion
NCT03452774	Artificial Intelligence trial for Matching and Registry	RECRUITING	All cancer types	Clinical Trial Matching	Y (non-specific)
